# Quantifying the impact on navigation performance in visually impaired: Auditory information loss versus information gain enabled through electronic travel aids

**DOI:** 10.1371/journal.pone.0196156

**Published:** 2018-04-26

**Authors:** Alex Kreilinger, Thomas Georgi, Gudrun Pregartner, Domagoj Ivastinovic, Tamara Pichler, Andrea Berghold, Michaela Velikay-Parel

**Affiliations:** 1 Medical University of Graz, Department of Ophthalmology, Graz, Austria; 2 Medical University of Graz, Institute for Medical Informatics, Statistics and Documentation, Graz, Austria; Center for Healthy Start Initiative, NIGERIA

## Abstract

This study’s purpose was to analyze and quantify the impact of auditory information loss versus information gain provided by electronic travel aids (ETAs) on navigation performance in people with low vision. Navigation performance of ten subjects (age: 54.9±11.2 years) with visual acuities >1.0 LogMAR was assessed via the Graz Mobility Test (GMT). Subjects passed through a maze in three different modalities: ‘Normal’ with visual and auditory information available, ‘Auditory Information Loss’ with artificially reduced hearing (leaving only visual information), and ‘ETA’ with a vibrating ETA based on ultrasonic waves, thereby facilitating visual, auditory, and tactile information. Main performance measures comprised passage time and number of contacts. Additionally, head tracking was used to relate head movements to motion direction. When comparing ‘Auditory Information Loss’ to ‘Normal’, subjects needed significantly more time (p<0.001), made more contacts (p<0.001), had higher relative viewing angles (p = 0.002), and a higher percentage of orientation losses (p = 0.011). The only significant difference when comparing ‘ETA’ to ‘Normal’ was a reduced number of contacts (p<0.001). Our study provides objective, quantifiable measures of the impact of reduced hearing on the navigation performance in low vision subjects. Significant effects of ‘Auditory Information Loss’ were found for all measures; for example, passage time increased by 17.4%. These findings show that low vision subjects rely on auditory information for navigation. In contrast, the impact of the ETA was not significant but further analysis of head movements revealed two different coping strategies: half of the subjects used the ETA to increase speed, whereas the other half aimed at avoiding contacts.

## Introduction

Pedestrian navigation is a serious challenge for people with low or no vision. A well-known strategy for compensation is to rely on other senses including tactile and aural [1]. For example, it is known that blind people evaluate echoes to gain information about their environment [2, 3]. Generally, hearing becomes more important for navigation as vision decreases. To draw conclusions about the magnitude of this beneficial effect, many research groups aimed to quantify the opposite, negative effect of dual sensory loss (DSL) [4–11]. However, the reported results obtained from questionnaires deviate between these studies. Some revealed that DSL has a significant negative impact on activities of daily living [5–9] whereas others did not find this effect [4, 10].

It is generally difficult to objectively measure navigation performance due to a lack of objective tools to quantify functional vision [12] as opposed to visual functions (e.g., visual acuity (VA) [13, 14], peripheral vision [15], or contrast sensitivity [16]). To measure changes in navigation performance between different modalities, functional vision tests need to present an adequate challenge. On the one hand, if a test is too difficult it is unlikely to measure small changes. On the other hand, a test too simple results in flooring effects with similar outcomes.

Some studies have aimed to quantify performance in navigation tasks. Unfortunately, most of these are not suitable for our research goal which involves only subjects in the low vision range, as illustrated by the following examples. In a study that aimed to compare mobility performance of visually impaired adults before and after orientation and mobility training [17], a main problem was the wide range of visual impairment (VA between 6/6 and 6/3000), which meant that the used mobility test could not have been challenging for each individual subject. The test was difficult as it comprised 100 obstacles, not all of them with high contrast, on a long course and it included additional tasks (placing objects) and a glare source to further increase the difficulty level. Likely as a result, the authors did not measure an improved mobility performance after subjects received training. Another example for a setup that may be too difficult for a low vision group was shown in [18], where subjects had to read arrow signs. Additionally, they were required to walk in an outdoor course where varying weather conditions and randomly appearing pedestrians increased the difficulty unpredictably. In contrast, tests with over-simplified tasks reduce the subjects’ dependency on visual information. Brown et al. [19] set up very simple paths (square, triangular, and slalom courses). Subjects knew which shapes they had to pass through and the only obstacles were poles to define the shapes of the courses. Other simplified tasks were used in a study to measure the effects of visual impairment on tasks of everyday life [20]. Mobility was represented by a 4-m walk, standing up from a chair, and ascending/descending stairs. Although these excercises can provide valuable information, they may not be entirely representative of how well people with low vision master more complex navigation tasks. Other issues arise when using settings that are not suitable for test-retest scenarios due to uncontrollable environments or inevitable learning effects when using only a single layout [21, 22].

Difficulties in designing a suitable obstacle course for measuring navigation performance are discussed comprehensively in Leat et al. [23]. The University College London (UCL) Pedestrian Accessibility and Movement Environment Laboratory (PAMELA) and the Graz Mobility Test (GMT) demonstrate possible solutions [24, 25]. Both of these maze-based mobility tests are challenging for low vision subjects, can be rearranged for multiple pass-throughs to avoid learning effects, have uniform lighting, do not require subjects to read signs, and are located in a restricted area. Although PAMELA’s main purpose is analyzing pedestrian transport situations, it was successfully used to test performance before and after gene therapy [24], whereas the GMT was specifically designed to quantify navigation abilities in candidates for artificial vision. A former study revealed that the GMT adequately challenges subjects in the low vision range [25] with a VA of 1.0 LogMAR or worse but is not suitable for subjects with better vision due to its high contrast obstacles. The GMT is equipped with cameras that can capture head movements. Head movements are not only used for detecting visual cues but can increase the accuracy of sound localization [26] and are involved in stabilization and anticipatory orientation mechanisms in walking [27].

The first goal of this study was to measure the impact of decreased information, represented by artificially induced auditory information loss, in low vision subjects. Although the GMT was located in a quiet room, self-generated sounds, such as interactions with obstacles and footfalls (mouth clicks for active echolocation were not permitted), as well as ambient noise from a close-by operator and traffic outside provided potential auditory cues for orientation. In contrast, the second goal was to examine the benefits of information gain. As a means to increase sensory information, we chose an ultrasound-based electronic travel aid (ETA), which provided tactile feedback by increasing vibration intensity inversely related to the distance between an obstacle and the ETA.

There are relevant publications that have already dealt with ETAs and auditory information for navigation in visually impaired people. However, these used different approaches to cater to their respective aims. Chebat et al. tested a tongue display unit [28] in congenitally blind subjects with no history of light perception and a sighted control group. They found that the blind subjects outperformed the control group. In a following study [29], a different ETA was used: the EyeCane, which gives acoustic and tactile feedback based on objects’ distance measured with infrared light. The authors showed that four groups (blindfolded congenitally blind, blindfolded low vision/late blind, blindfolded sighted, and sighted) performed at a level that was no longer significantly different after three sessions. However, in these sessions the subjects were able to learn the layout of the used mazes (real and virtual) which were not altered between trials. Recent publications from Kolarik et al. report about navigation performance related to auditory information and ETAs. First, they showed that blindfolded sighted subjects were able to use active echolocation via self-generated mouth click sounds [30]. In a short course, one single obstacle was placed randomly within a small margin or was absent. 85% of the obstacles were detected and 67% of the obstacles were successfully circumvented. A similar experiment was done with the Miniguide, an ETA based on ultrasound that emitted vibration signals inversely correlated to the distance between ETA and obstacle [31]. Blindfolded sighted subjects performed better with the ETA compared to echolocation as they managed to circumvent 93% of the obstacles. In a final study, both aspects were combined and tested with congenitally blind non-echolocators, one blind echolocator, and a sighted control group [32]. Three modalities were tested, again with the one-obstacle course: visual only (performed only by the sighted control group), auditory only (all subjects blindfolded), and ETA (all subjects blindfolded and wearing hearing protection). The main findings showed that congenitally blind were generally better in circumventing obstacles based on echolocation than the blindfolded control group. The authors argue that the question remains open whether this finding is true for late-onset blindness as well.

The studies from Chebat et al. and Kolarik et al. have in common that they investigated sensory influences as isolated as possible. They also showed that it is likely that sighted control groups behave differently than actual low vision/blind subjects. This assumption is further backed by a study that demonstrated early- and late-blind performed better than blindfolded sighted subjects in a haptic matching task [33], although blindfolded subjects showed greater learning rates [34, 35].

Our intention was not to compare the effects of auditory information loss or the usage of an ETA in relation to a sighted control group but to measure relative changes within individual low vision subjects. Therefore, our subjects provided their own baseline by being tested in the GMT in a modality that represented their normal navigation. Furthermore, the GMT is not designed for sighted subjects and we would only measure a flooring effect as subjects would not be challenged and just finish the test with their maximum walking speed and without contacts with obstacles. Our aim was to provide low vision subjects with a navigation task that is challenging and therefore able to measure changes in performance. Changes in performance for the worse can realistically be based on hearing deterioration in elderly low vision people. However, the performance can also change for the better if people learn to use assistive devices like ETAs that are appropriate for their needs and abilities.

It is a generally accepted fact that early-onset or congenitally blind people develop compensation mechanisms based on tactile and auditory senses [36]. There are fewer observations showing a similar compensation if blindness sets in later in life. Still, in Voss et al. it was shown that both late- and early-blind subjects were able to outperform a sighted control group in an auditory task [37]. We assume that such a compensation mechanism does not form spontaneously but rather develops gradually as people with low vision learn to rely on additional senses with their vision deteriorating over time. Therefore, we believe that a loss of auditory information in low vision subjects would lead to a decreased performance in terms of longer passage times and/or more undesired collisions with obstacles in the GMT. Furthermore, we expect an increase of head movements which are caused by a more expansive search for visual and auditory cues and by orientation losses. Inversely, we expect that information gain would lead to a better performance and a reduced amount of head movements. We base this assumption on the fact that walking through the GMT is enough of a challenge that additional information is of value to the subjects.

We thus aimed to address the following questions: How much does hearing loss affect performance and head movements? How much does the use of an ETA affect performance and head movements?

To answer these questions, we recorded and calculated these four endpoints: passage time, number of contacts, mean of the absolute relative viewing angle (MARVA), and percentage of orientation loss. The former two represent the main performance measures, the latter two are used for in-depth analysis of subjects’ behavior in the GMT.

## Materials and methods

### Subjects

Participants were included based on the following criteria: (i) VA ≥1.0 LogMAR; (ii) subjects declared to have no hearing impairment and were able to make normal conversation; (iii) subjects declared to have no walking impairment which was confirmed visibly. The study followed the tenets of the Declaration of Helsinki and was approved by the Ethics Committee of the Medical University of Graz, Austria. Informed consent was obtained from all subjects prior to enrolment. Ten subjects, aged 54.9±11.2 (mean±standard deviation) years, participated in the study; six of them were female. VA results determined via the grating acuity test (GAT) [16] ranged from 1.0 to >2.7 LogMAR. Visual field was measured with Goldmann perimetry and the anatomical visual field score (AVFS) was determined according to [38]. The main cause for low vision was retinitis pigmentosa (RP) but also subjects with uveitis, sympathic ophthalmia, and severe myopia were included in the study (Table 1).

**Table 1:** Subjects participating in the study.

ID	VA OS/OD [LogMAR]	AVFS OS/OD [%]	Cause for low vision	Age	Sex
1	1.4/1.3	30.7/18.4	RP	61	F
2	>2.7/>2.7	5.2/0.0	SO, AO	53	M
3	1.1/1.9	2.8/0.0	RP	49	F
4	>2.7/>2.7	0.0/1.9	RP	33	F
5	>2.7/>2.7	0.9/1.9	RP	51	M
6	>2.7/>2.7	0.0/0.0	RP	71	M
7	1.1/1.2	16.0/6.6	Uveitis	54	F
8	>2.7/>2.7	0.0/0.0	RP	51	F
9	1.6/1.0	1.4/1.4	RT, myopia	72	F
10	>2.7/>2.7	0.0/0.0	RP	54	M

Visual acuity (VA) for both eyes (left eye/right eye) and anatomical visual field score (AVFS) for the ten participants. Causes for low vision are retinitis pigmentosa (RP), sympathetic ophthalmia (SO) due to anophthalmus (AO), uveitis, myopia, and retinal tearing (RT).

### Setup

**Fig 1:**
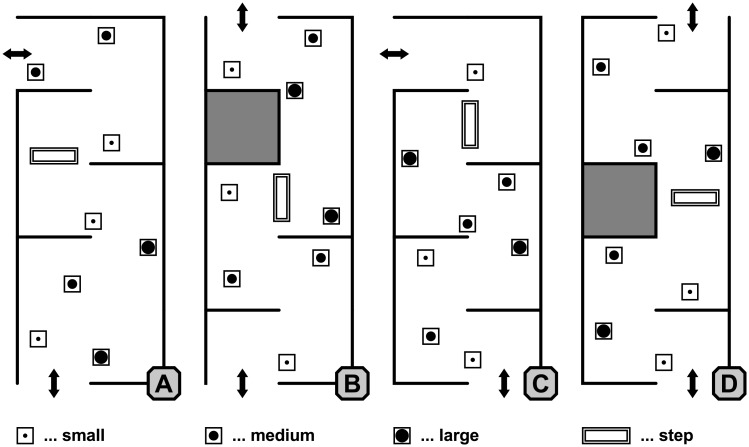
Graz mobility test. The schematic shows the four different maze variants A, B, C, and D. Obstacles (small, medium, large, and the step) and the walls can be repositioned without effort between runs. Arrows show the entrance and exit of each variant, depending on the walking direction.

The GMT consists of a 700×280 cm maze located in a quiet room in a low-frequented part of the eye hospital. Apart from occasional ambient sounds from the corridor or the street on the opposite side, no directional auditory cues are available. The maze is equipped with anti-glare lighting and is enclosed by 205 cm high walls covered with white tarp. The interior can be configured variably by suspending walls on the framework’s scaffold. Eight box obstacles of various heights (30×30×80/100/150 cm) and one step (97×43×17 cm) can be placed arbitrarily within the maze. The boxes are wrapped in matte black paper; the edges of the white step and the walls are covered with black duct tape in order to increase contrast. Four different maze variants were designed for the experiments (Fig 1). Variant A was only used for training purposes, whereas variants B, C, and D were used for the experiment in a pseudorandomized order.

### Procedure

In a first session, subjects were informed about the experiment whereupon they gave written consent. A grating test was performed to determine the VA and only subjects with a VA of ≥1.0 LogMAR were invited to session 2. Included subjects were then introduced to the ETA (“Ray—Ultrasonic Guide”, CareTec, Austria). The function of the ETA is based on ultrasonic waves that are reflected on surfaces within the range of the sonic beam (up to 2.5 m) and it gives feedback about the proximity to the detected surface by increasing the vibration intensity. A review on similar ETAs can be found in [39]. Subjects were particularly informed that the ETA is no substitute for a standard white cane, as objects on the ground are difficult to detect. Rather, the device is intended to serve as an additional tool for finding objects at the heights of the hips, shoulders, or the head. The ETA was given to the subjects for the duration of one week until the second session took place. They were asked to use the device in their known and safe environment for 1 h a day. Subjects were advised to point the ETA in front of them and scan their environment by swiping expansively. They were not forced to use a specific technique since each individual had one week to practice and find their preferred strategy.

The selected ETA was purely chosen as an example for providing the required gain of information. None of the authors are and were involved at any time with the ETA supplier and there are no potential conflicts of interest. In addition, we do not claim that the provided ETA represents the best individual solution for each participant.

One week later, the maze variants of the GMT were passed in three different modalities: i) ‘Normal’: without any mobility aid (including the white cane) or hearing protection, thereby making visual and auditory information available; ii) ‘Auditory Information Loss’: artificial hearing loss was precipitated by equipping the subjects with both ear plugs (single number rating, SNR = 37 dB) and earmuffs (SNR = 26 dB). Wearing the hearing protection, subjects could no longer listen to conversations at normal speaking volume and their available information was reduced to mostly visual only; iii) ‘ETA’: with the ETA which added tactile information to the already available visual and auditory information. To become acquainted with the GMT, subjects passed maze variant A once in each modality. For safety reasons, an operator would always stay within close proximity to the subjects. The actual test consisted of passing through the three remaining variants of the maze—B, C, and D—in the three modalities. The sequence was randomly generated in Matlab (The MathWorks, Inc., USA) in a way that each variant/direction combination was passed exactly once in each modality, resulting in a total of 18 runs (6 runs per modality). We used a pseudorandomization in order to guarantee that the maze was reconstructed after every two runs of alternating walking directions to minimize potential learning effects. Subjects were instructed to pass through the maze as fast as possible with as few contacts with objects and/or walls as possible while maintaining a safe walking speed. Passage time and number of contacts were explained to be of equal importance. During the run, the operator recorded the passage time and the number of contacts.

### Video analysis

Ten video cameras on top of the scaffold were used to record each run. On the floor of the maze, magenta-colored markers were affixed at equal spacings. Furthermore, the subjects had to wear a crown with five distinctly colored spheres. With the known positions and parameters of the cameras and the dimensions of the crown, it was possible to determine the position and orientation of the crown in each video frame. The calculation was based on the POSIT algorithm [40]; the evaluation scripts were modified from a previous study [41]. By consecutive analysis of each frame, the walking path could be reconstructed. In a further step, data were filtered with a median filter to remove outliers caused by false detections and smoothed to reduce discontinuities, which were, for example, caused by switching between cameras or swaying, as there seems to be a positive correlation between vision impairment and swaying [42, 43]. The motion direction in each frame was calculated by finding the vector between the current and the next position that was at least 60 cm away. This value was chosen to represent distances at the border of the reaching or peripersonal space [44]. Viewing directions were compared to the motion direction in each frame to obtain the relative viewing angle which illustrates how much the subject’s gaze deviates from the walking path.

### Endpoints

To measure the subjects’ performance through the maze, passage time and number of contacts were assessed for each run. Passage time was recorded as seconds between entering and exiting the maze; number of contacts comprised the number of times the subject touched an obstacle, the step, or a wall.

To analyze the subjects’ deviations from the walking path we devised a third measure: MARVA. Here, the absolute deviation from the walking path is averaged over every individual run. High MARVA values can either be caused by frequent orientation losses or by expansive scanning movements that are used as part of the subject’s navigation strategy. To specifically measure orientation loss, the percentage of time with a relative viewing angle above a predefined threshold was calculated for each run. This threshold was set to 120° as relative viewing angles of that magnitude are almost certainly related to orientation losses, whereas values up to 90° might also be caused by sidestepping.

### Statistical analyses

To assess differences between the modalities (‘Normal’ vs. ‘Auditory Information Loss’ and ‘Normal’ vs. ‘ETA’) for each of the four endpoints, we used linear mixed models (LMM) with subjects as random effects due to the repeated measurement design of the study. Even though we were only interested in differences between the respective modalities, the model included both modality and variant as fixed effects because the maze variant also constitutes a known source of variation. We also tested for interaction between these two factors but found that this term could be dropped from the model. For contacts, a Poisson model was also considered but is not presented here since it yielded similar results as the linear model. Data are presented as means for the reference modality ‘Normal’ and mean differences for the other modalities (each with 95% confidence intervals), as well as with boxplots.

In addition, we investigated the distribution of relative viewing angles of each subject to show individual head movement characteristics. A narrow distribution would indicate that the subject gazed mostly in the direction of the intended walking path whereas a broad distribution would be characteristic of subjects with more expansive head movements. Distributions with a high number of large angles represent subjects who frequently experience orientation losses. As an exploratory analysis it was thus investigated whether subjects whose passage times improved with ‘ETA’ showed different viewing patterns than subjects whose passage times were slower with ‘ETA’ (as compared to ‘Normal’). This grouping was achieved by comparing individual subjects’ median passage times for the two modalities. For the exploratory analysis, we used a mixed model for the endpoint MARVA considering only runs with modality ‘Normal’ or ‘ETA’. The model included all fixed and random effects from the main analysis but also contained group and an interaction term between group and modality as additional fixed effects. The same analysis was repeated with the endpoint percentage of orientation loss to find out whether differences are based on actually different viewing behaviors or rather on an increased number of orientation losses. Finally, to further analyze the differences between groups, we investigated the correlations between passage times and number of contacts within each modality (‘Normal’ or ‘ETA’) and group. Individual runs were pooled by their median and Spearman correlation coefficients were calculated. Mixed model analyses were performed using R version 3.2.2 (packages nlme and lme4) and SAS version 9.4 (proc MIXED). A p value of <0.025 was considered statistically significant due to separate tests for the modalities ‘Auditory Information Loss’ and ‘ETA’ respectively.

## Results

**Fig 2:**
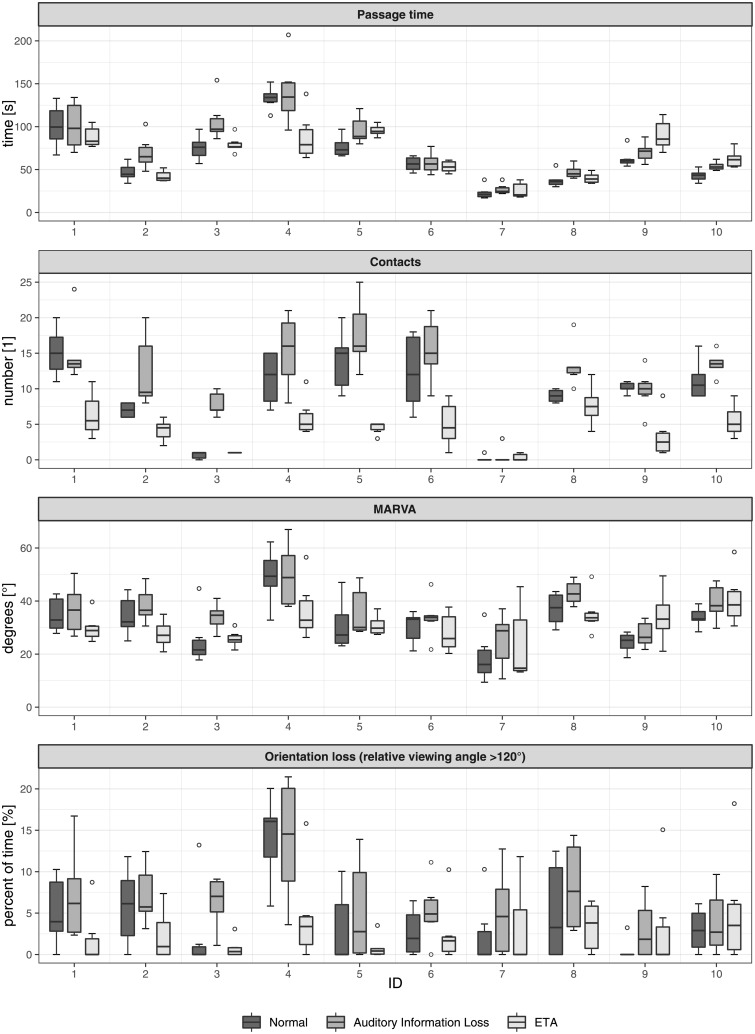
Endpoints. The four measured endpoints (Passage time, Contacts, MARVA, and Orientation loss) in the three modalities (‘Normal’ with visual and auditory information available, ‘Auditory information loss’ with only visual information, and ‘ETA’ with tactile, visual, and auditory information) for each subject.

The performance of the individual subjects for each of the four endpoints are shown in Fig 2.

### Passage time

The mean passage time with modality ‘Normal’ was 65.6 s (95% confidence interval [47.2, 84.0]). On average, subjects needed 11.4 s longer with ‘Auditory Information Loss’ ([5.2, 17.6], p<0.001) and 0.8 s longer with ‘ETA’ ([-5.3, 7.0], p = 0.790).

### Number of contacts

The mean number of contacts with modality ‘Normal’ was 9.1 ([6.6, 11.7]). There were on average 3.0 more contacts with ‘Auditory Information Loss’ ([1.8, 4.2], p <0.001) and 4.7 fewer contacts with ‘ETA’ ([-5.9, -3.5], p<0.001).

### MARVA

The mean MARVA with modality ‘Normal’ was 31.9° ([27.6, 36.1]). It was on average 4.5° higher with ‘Auditory Information Loss’ ([1.7, 7.4], p = 0.002) and 0.6° smaller with ‘ETA’ ([-3.4, 2.2], p = 0.678).

### Percentage of orientation loss

The mean time of orientation loss with modality ‘Normal’ was 4.4% ([2.6, 6.2]). With modality ‘Auditory Information Loss’ it was about 2.1% higher ([0.5, 3.7], p = 0.011) and with modality ‘ETA’ it was on average 1.6% lower ([-3.2, 0.0], p = 0.051).

### Further exploratory analysis

**Fig 3:**
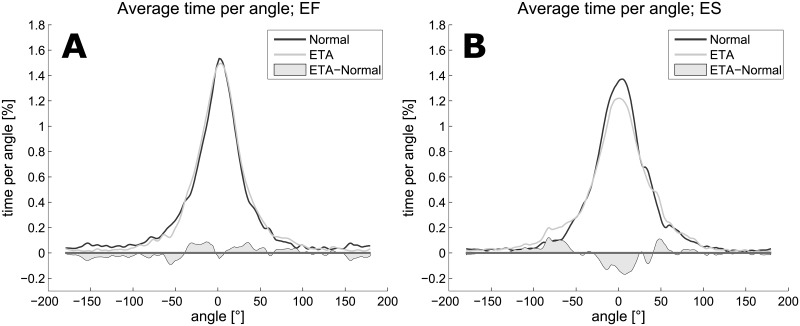
Angle distributions for the two groups EF (A) and ES (B). The different behaviors of the two groups are reflected in the width of the distributions when comparing ‘Normal’ to ‘ETA’. Subjects who were faster with ‘ETA’ had narrower distributions which indicates that they spent more time in the lower angle range. The opposite effect can be observed in the ES group.

By grouping the subjects by whether their median passage time was shorter with ‘Normal’ (group ‘ETA’ slower, ES) or ‘ETA’ (group ‘ETA’ faster, EF) we obtained the following classification: EF: subjects 1, 2, 4, 6, and 7; ES: subjects 3, 5, 8, 9, and 10. As a purely exploratory and descriptive analysis we pooled all angles from runs with modalities ‘Normal’ or ‘ETA’ to investigate the distribution of these angles. Fig 3 shows differences between the groups EF and ES: subjects in group EF had a narrower distribution in ‘ETA’ compared to ‘Normal’; subjects in group ES had a wider distribution in ‘ETA’ compared to ‘Normal’.

This observation is further substantiated by a significant interaction between modality and group in the LMM used to address this research question for MARVA (p = 0.010). For percentage of orientation loss this interaction was not significant (p = 0.062). Correlation analyses between passage time and number of contacts showed that, whereas the two performance measures were strongly positively correlated in group EF (r = 0.82, p = 0.089 for ‘Normal’; r = 0.97, p = 0.005 for ‘ETA’), they were negatively correlated in group ES (r = -0.10, p = 0.950 for ‘Normal’; r = -0.46, p = 0.434 for ‘ETA’).

## Discussion

In current literature the assessment of general functional vision is mostly based indirectly on visual function tests or on questionnaires [7, 10, 13–15, 45–47]. Visual function tests can only assess single aspects of vision (VA, VF, or contrast sensitivity) and it is impossible to deduce general vision-based performance by means of these tests. The aptitude of questionnaires to objectively quantify navigation skills remains questionable; both high correlations between self-reported capabilities and clinical findings [48] but also disconnects between self-reported difficulties and perceived/assessed difficulties [49] are reported in literature. There are noteworthy examples in literature that directly test visual function. However, these are often not applicable for general assessment as they are based on measures that are designed to test specific applications or devices, such as path efficiency while using an indoor navigation system that requires placement of radio frequency identification (RFID) tags [50] or the route finding time while using a digital sign system [51]. Only a few tests, such as the GMT and PAMELA, can be used to quantify general navigational performance for different modalities in terms of measures like passage time and number of contacts. Additionally, the GMT records the position and orientation of the subjects’ heads. These data can be analyzed in order to obtain in-depth information about subjects’ coping strategies during navigation. In fact, this method enabled us to detect different reactions to information loss and gain.

### Passage time

The passage time was expected to increase when less information was provided to the subjects and to decrease with additional information. Surprisingly, this hypothesis was not corroborated entirely. While subtraction of information in form of modality ‘Auditory Information Loss’ lead to significantly longer passage times, additional information in ‘ETA’ did not cause shorter passage times on average, both compared to ‘Normal’. Nevertheless, it is an important finding that auditory information loss had a negative effect on performance based on passage time. It indicates that subjects were challenged by the GMT; otherwise the decrease of sensory information other than vision would not have had an effect. This is in line with our hypothesis that people with low vision rely on additional senses for navigation. The fact that passage time was not decreased on average in modality ‘ETA’ may have been based on varying behavior patterns. This latter finding will be addressed in more detail later on.

### Number of contacts

In contrast to passage time, our hypothesis that more information leads to fewer errors and less information leads to more errors could be corroborated in terms of number of contacts. No matter if the subjects were faster or slower when using the ETA, they always benefited by making fewer contacts with obstacles or walls. ‘Auditory Information Loss’ had the opposite effect: on average, subjects made significantly more errors when their hearing was dampened. These changes in performance for both better and worse clearly demonstrate that the difficulty level of the GMT is such that low vision subjects can either benefit from getting more information based on assistive devices, such as an ETA, but also be impeded by losing further information, which was in our case facilitated by ‘Auditory Information Loss’.

### MARVA

The MARVA is a performance measure based on the relative viewing angle and gives insight on how subjects orient themselves in different modalities. Its properties are similar to passage time: the MARVA was significantly higher in ‘Auditory Information Loss’ compared to ‘Normal’ but a significant difference between ‘ETA’ and ‘Normal’ was not found. Apparently, subjects who need more time in the maze also have higher deviations of viewing angles from the walking path. We provide several possible explanations for an increased MARVA. First of all, in ‘Auditory Information Loss’, subjects can no longer make use of auditory cues and have to rely more on their remaining vision. However, because they have a limited visual field, more scanning movements might be necessary. Second, it might be possible that head movements are attempts to locate sounds more accurately, as it was shown that head movements can be beneficial for localization [26]. The third and most likely explanation for an increased MARVA, however, is frequent orientation loss. The individual cause of an enlarged MARVA can be verified by examining a subject’s relative viewing angle distribution: it indicates whether the larger MARVA is caused by a higher percentage of high angles or by a generally broad distribution of angles. In addition, the fourth and last endpoint aims to specifically target orientation losses.

### Percentage of orientation loss

To specifically measure the impact of orientation loss on the MARVA we considered the percentage of time above a relative viewing angle of 120°. Orientation losses were significantly more frequent in ‘Auditory Information Loss’ compared to ‘Normal’. There was no significant difference between ‘ETA’ and ‘Normal’. A significant decrease of orientation loss in ‘ETA’ would have been a strong indication that using assistive devices is beneficial, even if they are used differently. One reason for not observing such a decrease might have been that although subjects were able to avoid collisions with objects in their immediate proximity, they did not gain more information about the general layout of the maze. Therefore, it was still possible to get lost within the maze even though there were fewer contacts with obstacles.

### Exploratory further analysis

When comparing ‘ETA’ to ‘Normal’, significant differences were found only in the number of contacts. This finding induced a more thorough analysis to look for potential reasons. We discovered that only half of the subjects became faster using the ETA (group EF) whereas the second half became slower (group ES). This separation indicates that subjects rely on diverse coping strategies when provided with a new assistive device. The correlations between passage time and number of contacts were different between the two groups. Subjects in group EF displayed a large positive correlation between passage time and number of contacts (for both ‘Normal’ and ‘ETA’), whereas the correlation was negative in group ES. This finding points towards two different strategies, depending on how much subjects relied on the device: (i) a “daring” strategy where subjects fully relied on the mobility aid as an additional sensor: these subjects were able to use the device in the most efficient way by decreasing the passage time and also the number of contacts in the process; (ii) a “cautious” strategy in which subjects took their time but in turn emerged from the maze with fewer errors. As both passage time and number of contacts were introduced to the subjects as equally important, these results reflect the subjects’ interpretations or personal preferences. Our exploratory analysis using LMM showed a significant interaction between group and modality for MARVA but not for percentage of orientation loss. Therefore, the difference is indeed based on different coping strategies and not just on the fact that one group loses orientation more often than the other. We also investigated the distribution of relative viewing angles over time. Expecting a normal distribution with mean 0, we were interested in whether the spread of the distribution varied between the two groups EF and ES for the modalities ‘Normal’ and ‘ETA’. Such differences could indicate whether subjects were more inclined to use head movements, thereby not fully trusting the mobility aid, or to keep the head pointing forward, relying heavily on the mobility aid. These differences can be observed in Fig 3. It is noticeable that the distribution of group EF is narrower (relatively more time with small angles) in ‘ETA’ compared to ‘Normal’. The opposite is true for group ES. These primary personal preferences might be alterable through prolonged training. However, testing for different strategies before assigning an assistive device may help in deciding which device is best suited for each individual. Furthermore, it is possible to monitor and quantify changes in usage over time.

### Limitations

A general limitation of the study was the low number of subjects and the high variability of remaining visual function. Impairments that cause low vision are diverse, as are the subjects’ different coping strategies. However, the GMT was designed as a measurement tool capable of documenting and analyzing differences not only between individuals but also within one single person over time. Additionally, using VA as inclusion criteria was not optimal, as visual field was shown to be a better predictor of poor mobility performance [52]. However, all of the included subjects had severely restricted visual fields. Finally, the training period of one week might have been too short for some of the subjects to become acquainted with the ETA.

Further design choices might be considered limitations, for example not using a sighted control group. However, we were only interested in relative changes with each individual providing their own baseline via the ‘Normal’ modality. Moreover, testing sighted subjects is not advised in the GMT due to flooring effects for people with better than low vision and we tried to avoid blindfolding a sighted control group as it seems likely that they behave differently than blind or low vision subjects [28, 32–35]. Nevertheless, it would have been interesting to measure if and to what extent low vision subjects rely on auditory information more than sighted subjects. The current study was not designed to investigate this topic. Therefore, it remains an open question for future experiments.

A possible limitation might have been that subjects were not instructed on how exactly to hold and perform navigation tasks with the ETA, as was done in [31]. However, it is possible that the differences in behavior would not have been discovered if subjects had not have had the freedom of choice on how to use the ETA. Also, preventing subjects from using the ETA how they preferred might have been inconvenient and resulted in a negative impact on performance.

## Conclusion

Our study showed that the GMT is capable of quantifying effects of sensory information loss versus gain in low vision subjects. A loss of auditory information resulted in significant changes for the worse in all measured endpoints when compared to ‘Normal’. Finding evidence that performance decreases with reduced sensory information has important implications: it means that subjects rely on auditory information before reaching a state of total blindness. With this knowledge, affected people can prepare by incorporating auditory information in their navigation training or by obtaining information on hearing aids. They might even consider using active echolocation which can even be learned by sighted people [30]. If this is not possible, they might train using other sensory information, e.g. tactile. The use of an ETA only showed significant results in reducing the number of contacts. However, by further analyzing head movements we were able to illustrate and measure varying behavioral patterns in navigation. We identified two different coping strategies: one half of the subjects used a “cautious” strategy and mainly reduced contacts, whereas the other half used a “daring” strategy and increased their walking speed. Knowing how these devices help low vision subjects is crucial for providing the proper equipment and/or training to maximize their benefits.

## Supporting information

S1 File This excel file contains passage times, number of contacts, MARVA, and percentages of orientation loss for all subjects. (XLS)
